# Giant Serous Cystadenoma of the Pancreas (*⩾*10 cm): The Clinical Features and CT Findings

**DOI:** 10.1155/2016/8454823

**Published:** 2016-08-16

**Authors:** Qing-Yu Liu, Jun Zhou, Yu-Rong Zeng, Xiao-Feng Lin, Jun Min

**Affiliations:** ^1^Department of Radiology, Zengcheng People's Hospital, 1 Guang Ming Dong Road, Zengcheng, Guangdong 511300, China; ^2^Department of Radiology, Sun Yat-sen Memorial Hospital, Sun Yat-sen University, 107 Yan Jiang Xi Road, Guangzhou, Guangdong 510120, China; ^3^Department of Gastrointestinal Surgery, Sun Yat-sen Memorial Hospital, Sun Yat-sen University, 107 Yan Jiang Xi Road, Guangzhou, Guangdong 510120, China; ^4^Department of Hepatobiliary Pancreatic Surgery, Sun Yat-sen Memorial Hospital, Sun Yat-sen University, 107 Yan Jiang Xi Road, Guangzhou, Guangdong 510120, China

## Abstract

*Purpose*. To report the clinical features and CT manifestations of giant pancreatic serous cystadenoma (≥10 cm).* Methods.* We retrospectively reviewed the clinical features and CT findings of 6 cases of this entity.* Results.* All 6 patients were symptomatic. The tumors were 10.2 cm–16.5 cm (median value, 13.0 cm). CT imaging revealed that all 6 cases showed microcystic appearances (*n* = 5) or mixed microcystic and macrocystic appearances (*n* = 1). Five patients with tumors at the distal end of the pancreas received distal pancreatectomy. Among these 5 patients, 2 patients underwent partial transverse colon resection or omentum resection due to close adhesion. One patient whose tumor was located in the pancreatic head underwent pancreaticoduodenectomy; however, due to encasement of the portal and superior mesenteric veins, the tumor was incompletely resected. One patient had abundant draining veins on the tumor surface and suffered large blood loss (700 mL). After 6–49 months of follow-up the 6 patients showed no tumor recurrence or signs of malignant transformation.* Conclusions.* Giant pancreatic serous cystadenoma necessitates surgical resection due to large size, symptoms, uncertain diagnosis, and adjacent organ compression. The relationship between the tumors and the neighboring organs needs to be carefully assessed before operation on CT image.

## 1. Introduction

Pancreatic serous cystadenoma is a common type of pancreatic cystic tumor and accounts for 10–15% of all cases of pancreatic cystic tumor and 1-2% of primary pancreatic tumors [1]. These tumors are often small at diagnosis, 31 mm (1–238 mm) on average [2]. Patients usually (61%) do not exhibit relevant clinical symptoms but may develop symptoms as the tumors grow larger [2]. Because most of these tumors display benign biological behaviors, asymptomatic patients with small tumors are often recommended for conservative treatment [2]. Giant pancreatic serous cystadenoma (diameter ≥ 10 cm) is very rare, and only sporadic cases have been reported [3–11]. In this study, we examined the clinical features and CT findings of 6 cases of giant pancreatic serous cystadenoma. To our knowledge, this is the largest series being reported.

## 2. Materials and Methods

### 2.1. Patients

We searched clinical data of Sun Yat-Sen Memorial Hospital, Sun Yat-Sen University, between January 2003 and December 2015, and a total of 57 cases of pancreatic cystic tumors that were pathologically confirmed were found, including 28 cases of serous cystadenoma, of which 6 (10.5%) were giant pancreatic serous cystadenoma (diameter ≥ 10 cm). This retrospective study, which did not require consent from the patients, was approved by the ethics committee of the hospital.

Before operation, all 6 patients received abdominal 64-slice spiral CT examination (Sensation 64, Siemens Medical Solutions, Erlangen, Germany), including plain scans and triphasic enhanced scans. A nonionic contrast agent (Iopromide, 370 mgI/mL, Schering, Erlangen, Germany) was administered via the antecubital vein using a pressure injector at 3.5 mL/s with a dose of 1.5 mL/kg. Using the bolus tracer function, arterial phase images were collected 10 sec after the intensification threshold of the celiac level aorta reached 100 HU. Portal phase images and delayed phase images were collected 15 and 90 sec after the completion of arterial phase scanning, respectively. The slice thickness of CT image was 3.0 mm. CT angiography (CTA) was accomplished at the CT postprocessing workstation.

### 2.2. The Clinical Features and CT Findings Assessment

The electronic clinical data of these patients were retrospectively analyzed, including gender, age, symptoms, relevant laboratory examination results (e.g., AFP, CEA, CA125, CA199, TDIL, and DBIL), surgical approach, and prognosis.

Two abdominal radiologists retrospectively evaluated the CT images using the picture archiving and communications system. The radiologists were both aware that the 6 patients were diagnosed with pancreatic tumor but did not know other information, including pathological data. Image reviews were done jointly and by consensus. The following CT findings were determined: tumor location, size, shape, capsule, calcification, morphological pattern, enhancement pattern, pancreatic or bile duct dilatation, and other relevant observations. Based on their morphological patterns, the tumors were classified as microcystic, macrocystic (or oligocystic), mixed microcystic and macrocystic, and solid type [2]. The density of pancreatic masses was compared with adjacent normal pancreatic tissue and divided into hypodensity, isodensity, or hyperdensity.

## 3. Results

### 3.1. The Clinical Properties of Giant Pancreatic Serous Cystadenoma

The clinical characteristics of the 6 patients with giant pancreatic serous cystadenoma are summarized in Table 1. The patients were aged between 48 and 68 years (median age: 64 years) and included 3 females and 3 males. All patients exhibited symptoms, primarily abdominal bloating, abdominal pain, palpable mass, or vomiting. Laboratory examination (e.g., AFP, CEA, CA125, CA199, TDIL, and DBIL) showed no abnormalities except in 1 patient (case 4), who had concurrent gastric stromal tumor and rectal adenocarcinoma and presented with an elevated CEA level of 231 ng/mL (normal: 0–5 ng/mL). In this patient, the common bile duct was surrounded and compressed by the tumor and exhibited slight elevation of TBIL and DBIL levels (33.8 *μ*mol/L and 14.16 *μ*mol/L, resp.; normal ranges for these are 3.4–17.1 *μ*mol/L and 0–3.4 *μ*mol/L, resp.). All six patients were operated on due to uncertain diagnosis (*n* = 4), symptoms (*n* = 6), large size (*n* = 6), and adjacent organ compression (*n* = 1).

The pancreatic tumors were 10.2–16.5 cm in size (median, 13.0 cm). In 5 patients, the tumors were located at the body-tail of the pancreas; the patients therefore received distal pancreatectomy (4 patients also underwent splenectomy). Among these 5 patients, 2 patients (case 5 and case 2) underwent concurrent partial transverse colon resection or omentum resection due to tight adhesion. One patient (case 4) whose tumor was located in the pancreatic head underwent pancreaticoduodenectomy, but the resection was incomplete because of encasement of the portal and superior mesenteric veins; this patient also underwent portal vein repair because of the intraoperative portal vein injury. One patient (case 2) had apparent draining veins on the tumor surface and suffered large blood loss (700 mL); 1 patient (case 5) received concurrent resection of colon cancer and gastric stromal tumor and had a blood loss of 3,000 mL; the other 4 patients had blood loss of 100–200 mL during operation. Among all 6 patients, there was 1 case of postoperative infection and 1 case of mild pancreatic leak. After 6–49 months of postoperative follow-up, the 6 patients showed no tumor recurrence, signs of malignant transformation, or signs of pancreatic compromise.

**Figure 1:**
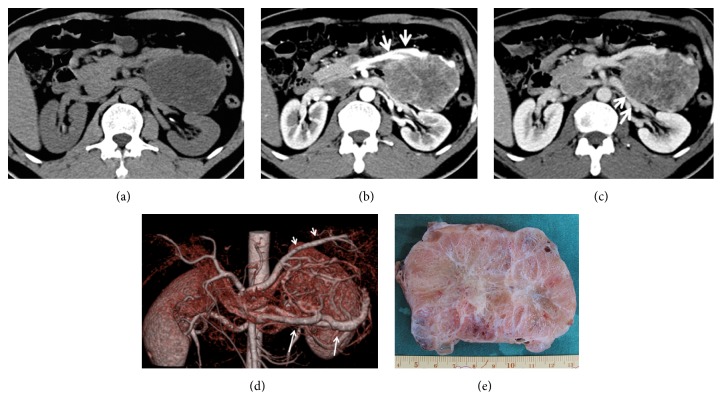
A 48-year-old male patient with a giant serous cystadenoma of the pancreas. (a) A low-density pancreatic tumor was noted on CT plain scan. (b) The tumor showed early enhancement with abundant draining vein (arrow) on arterial phase image. (c) The tumor showed honeycomb-like shapes with decreased enhancement on portal phase image. Left renal vein compression was noted (arrow). (d) The feeding splenic artery (short arrow) and draining veins (long arrow) were showed on CT angiography (CTA). (e) The tumor specimen was honeycomb-like appearance with central scars.

**Figure 2 fig2:**
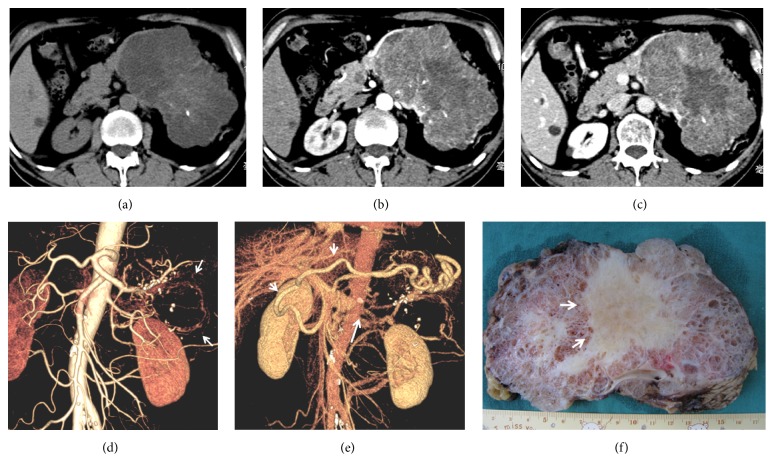
A 67-year-old male patient with a giant serous cystadenoma of the pancreas. (a) A low-density, lobulated tumor with dotted calcification and isodense central scar was noted on CT plain scanning. (b) The tumor showed early enhancement on arterial phase image. (c) The honeycomb-like tumor displayed decreased enhancement on portal phase image with a hypodense central scar. (d) CTA showed the feeding splenic artery and draining veins (arrow) on the tumor surface. (e) Portal phase vascular reconstruction showed left gastric vein varices (short arrow) and splenic vein stenosis (long arrow). (f) Tumor specimens displayed a honeycomb-like appearance with abundant central scar.

The tumors of the 6 patients generally had clear boundaries without capsule. The gross specimens manifested as honeycomb-like shapes (*n* = 5) or honeycomb-like shapes with macrocysts (*n* = 1). The tumor was composed of numerous tiny thin-walled cysts (averaged 1–12 mm in diameter) filled with serous fluid. In addition, the tumors had stellate fibrous scars in the center (Figures 1 and 2). Serous cystadenoma was pathologically confirmed without any malignant traits.

### 3.2. CT Findings

**Figure 3:**
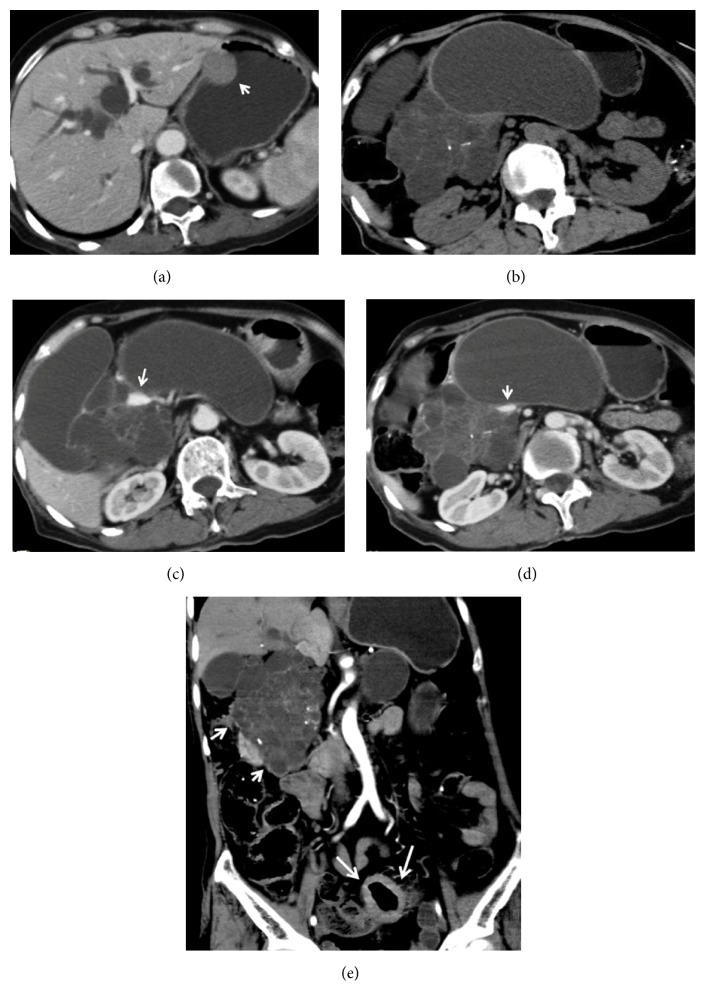
A 68-year-old female patient with a giant serous cystadenoma of the pancreatic head, concurrent colorectal cancer, and gastric stromal tumor. (a) Intrahepatic bile duct dilation and a gastric stromal tumor (arrow) were detected on CT plain scanning. (b) The pancreatic head tumor showed honeycomb-like appearance with multiple macrocysts and punctuate calcification. ((c) and (d)) Encasement of the portal and superior mesenteric veins (arrow) was noted on portal phase image. (e) The coronal CT image showed a pancreatic head tumor (short arrow) and rectal mass (long arrow).

The tumors of all 6 patients displayed lobulated appearance without capsule. Central calcification was showed in 5 patients. The morphological pattern of the tumor was microcysts (*n* = 5) or mixed microcystic and macrocystic type (*n* = 1). The tumors were of low density with central scars on precontrast CT scanning. The septa of cysts and central scars showed early enhancement on arterial phase images and decreased enhancement on the portal phase images, manifesting rapid wash-out enhancement pattern (Figures 1 [Fig fig2]–3). Cholangiectasis (*n* = 1) (case 4), encasement or compression of splenic arteries and veins (*n* = 6), adhesion to the stomach wall (*n* = 2), compression of left renal vein (*n* = 2), and adhesion to the transverse colon (*n* = 1) were noted on CT image. The tumor was located at the pancreatic head in 1 case (case 4), which showed encasement of the portal vein, superior mesenteric vein, and common bile duct. The apparent feeding arteries and draining veins on the surface (*n* = 3) and concurrent regional portal hypertension (*n* = 2) were clearly revealed on CT angiography (CTA) (Table 1). The 6 patients exhibited no signs of metastasis at diagnosis.

## 4. Discussion

As imaging technologies improve and are more widely applied, the discovery of asymptomatic cystic tumors of the pancreas has increased. Pancreatic serous cystadenoma is a common type of benign pancreatic cystic tumor and is different from other types of pancreatic cystic tumors (e.g., mucinous cystadenoma and intraductal papillary mucinous neoplasms) that have overt or potential malignancy [12]. Pancreatic serous cystadenoma is typically found in women (accounting for 74% of cases) with a mean age of 58 years (16–99 years), and the tumor is usually located at the body-tail of the pancreas [2]. Giant serous cystadenoma (diameter ≥ 10 cm) is rare and accounts for 10.5% of surgically treated cases of pancreatic cystic tumors in our hospital. We herein retrospectively analyzed cases of giant pancreatic serous cystadenoma that were treated in this hospital as well as previously reported cases in English literatures between 2005 and 2015 (Table 1). The results showed that this entity was more likely to occur in women (76.5%, 13/17) with a mean age of 65.5 years and that pancreatic head involvement (58.8%, 10/17) was slightly more common than the other pancreatic areas.

Pancreatic serous cystadenomas are typically small, with a mean diameter of 31 mm [2]. During the follow-up period, the tumors maintained a stable size (57%) or grew slowly (37%, 0.4 cm/year) [2]. Due to their small size and slow growth, the tumors exerted almost no influence on neighboring tissues; therefore, the patients generally did not develop symptoms. However, giant pancreatic serous cystadenomas tend to adhere to, compress, encase, or even infiltrate into surrounding organs due to their large size. Consequently, the patients (70%) are likely to develop symptoms [2, 4], which mainly include abdominal pain, pancreatic-biliary symptoms, and other symptoms (abdominal mass, asthenia, nausea, and vomiting) (Table 1). Obstructive jaundice is very rarely reported [13]. Laboratory examination of giant pancreatic serous cystadenoma usually finds no elevated levels of tumor markers unless the tumors develop malignant transformation or the patient has concurrent malignant tumors at other sites.

Morphologically, pancreatic serous cystadenoma can be classified as microcystic, macrocystic (or oligocystic), mixed microcystic and macrocystic, and solid type [2, 14]. During follow-up visits, the tumors can change morphologically (e.g., microcystic type changes to macrocystic type, macrocystic type changes to mixed type, or macrocystic type changes to microcystic type) [1]. Microcystic (45%) and macrocystic types (32%) are most common patterns; the next is mixed (18%) and solid (5%) types [2]. Giant pancreatic serous cystadenoma predominantly exhibits the microcystic type (58.8%, 10/17), followed by macrocystic (or oligocystic) type (35.3%, 6/17) and mixed type (5.9%, 1/17) (Table 1). Giant pancreatic serous cystadenoma showed clear solitary mass with lobulated appearance on CT images. The microcystic type was characterized by numerous tiny cysts (subcentimeter) and a honeycomb appearance. However, the macrocystic (or oligocystic) type featured multiple large cysts (>2 cm) or unilocular cyst. The mixed type had the characteristics of both microcystic type and macrocystic type. These cysts of serous cystadenoma were divided by fibrous septa that can coalesce into a central scar. The septa or scars may exhibit calcification [14–16]. The fibrous septa displayed a rapid wash-out enhancement pattern on enhanced CT scanning. Although calcifications with a central scar resembling sun explosions are pathognomonic for pancreatic serous cystadenoma on CT examination, this sign is not usually detected [9] and has not been found in cases of giant serous cystadenomas reported by us or others (Table 1). It is necessary to differentiate pancreatic serous cystadenoma from other cystic tumors (e.g., mucinous tumors or intraductal papillary mucinous neoplasms) on CT exam, particularly macrocystic (or oligocystic) type and mixed type [12, 14]. Pancreatic serous cystadenoma of macrocystic (or oligocystic) type manifests as lobulated polycystic (or unilocular) lesions, whereas pancreatic mucinous tumor is characterized by a smooth boundary without a lobulated contour, an enhanced thick wall, and peripheral calcification. Furthermore, intraductal papillary mucinous neoplasms manifest as either pleomorphic or a clubbed, fingerlike cystic masses that connect to the dilated pancreatic duct [12, 14]. Endoscopic ultrasonography can not only clearly reveal the internal structure of tumors, such as dense microcystic clusters but also guide fine needle aspiration biopsy; therefore, this technique is valuable for diagnosis of pancreatic serous cystadenoma [1].

Although pancreatic serous cystadenoma is generally considered a slow-growing benign tumor, symptomatic or giant serous cystadenoma has been proposed to exhibit a high risk of malignant potential [1]. Strobel et al. reported that 3% of serous cystadenoma cases were malignant [17]. However, in the multinational study which was reported by Jais et al. [2], only three serous cystadenocarcinomas (0.1%) were recorded. Most reported cases of malignant serous cystic neoplasms had tumor sizes of greater than 10.0 cm [2, 18–23]. Among 10 cases of pancreatic malignant serous cystic neoplasms reported by Matsumoto et al. [18], 8 had tumor diameters greater than 10 cm, indicating that serous cystadenomas might acquire malignant potential according to the extent of growth. Although the diameter of malignant serous cystadenoma was usually larger compared with nonmalignant group, there is no report describing the correlation between size and malignancy in serous cystadenomas due to paucity of cases. Malignant serous cystic neoplasms exhibit a low level of malignancy and indolent clinical course. The preoperative diagnosis of malignant serous cystic neoplasms remains challenging. Currently, the diagnosis of malignant serous cystic neoplasms relies pathologically on distant metastasis beyond pancreatic and peripancreatic bed [2] or apparent local filtration or lymphatic metastasis [18, 19]. Organs that are infiltrated locally by malignant serous cystic neoplasms include the spleen (8%), small intestine (4%), stomach (4%), adrenal gland (4%), and microscopic blood vessels/nerve infiltration; the organs exhibiting metastasis include (in order of frequency) the liver (35%), followed by local lymph nodes (12%), bone marrow (4%), and lung (4%) [19, 21].

Treatment for pancreatic serous cystadenoma remains controversial. It is suggested that surgery should be recommended for uncertain cystic tumor (i.e., cases without definitive diagnosis of serous cystadenoma), symptomatic serous cystadenoma (diameter ≥ 4.0 cm), or tumors with rapid growth (annual growth of greater than 4 mm). However, small or asymptomatic pancreatic serous cystadenoma should be provided with conservative treatment and close follow-up [2, 15, 24]. For cases of giant serous cystadenoma, surgery is recommended due to large size, symptoms, uncertain diagnosis, and adjacent organ compression. The relationship between a giant serous cystadenoma and the neighboring organs, blood vessels, and bile duct is critical for determining tumor resectability. Giant pancreatic serous cystadenomas may develop tight adhesion to the colon [4, 6] and omentum; thus, concurrent removal of these structures is required. Patients with the portal vein, superior mesenteric vein encasement, or adherence are subjected to vascular resection and reconstruction [7] or incomplete resection with the blood vessels preservation. Laparoscopic fenestration or dome resection with chemocautery is considered for patients with macrocystic (or oligocystic) type tumors [3, 11]. For patients who are ineligible for major operations (e.g., elderly or high-risk individuals), palliative surgery also achieves sound outcome with low mortality and low morbidity [4] (Table 1). If the tumor has abundant feeding arteries or draining veins, the patient may be at great risk of intraoperative massive hemorrhage, which can be prevented by preoperative embolization of the feeding artery [7].

## 5. Conclusion

Due to their large size, giant pancreatic serous cystadenomas usually are symptomatic. Although this type of tumor displays characteristic findings on CT scanning, it is necessary to differentiate this tumor from other pancreatic cystic tumor types. In general, giant pancreatic serous cystadenomas require surgical excision, and the relationship between the tumor and the neighboring organs, blood vessels, and bile duct is critical for determining tumor resectability.

## Figures and Tables

**Table 1:** Clinical and CT manifestations of giant pancreatic serous cystadenoma.

Authors	Gender/age (y)	Symptoms	Size (cm)	Location	Morphological patterns	Calcification	CTA or DSA	Relationship with neighboring organs	Surgical procedure	Follow-up and outcome
Sakata et al. [3]	F/71	No	13.9	Head	Oligocystic type	No	Stretching of the adjacent vessels	NA	Dome resection with chemocautery using 100 mg minocycline hydrochloride	No postoperative complications and survived after 12 months of follow-up

Schulz et al. [4]	F/70	Abdominal discomfort with vomiting and lost weight	17.0	Head	Microcystic type	Yes	NA	Compression of the vena cava, the aorta, left liver lobe, and transverse colon. Involvement of the SMV and PV leading to severe portal hypertension	Right-sided hemicolectomy without tumor resection	Alive after 13 years of follow-up, symptoms are worsening and tumor is growing larger

Salemis and Tsohataridis [5]	F/83	General fatigue, epigastric pain, and weight loss	23.0	Head	Macrocystic type	No	NA	NA	Roux-en-Y cystojejunostomy	Alive after 13 years of follow-up, asymptomatic

Vernadakis et al. [6]	F/66	No	26.0	Head	Microcystic type	No	NA	Surrounding the right colonic vessels and compressing the IVC	Pylorus-preserving pancreaticoduodenectomy with a right hemicolectomy	Alive without postoperative complications

Tajima et al. [7]	F/72	No	13.0	Head	Microcystic type	No	Feeding arteries including GDA, RGA, SA, DPA, and IPDA Enlarged draining veins on the surface (drainage into the PV and SMV)	Tightly adherent to the SMV and PV	Preoperative embolization of the tumor-feeding arteries, pancreaticoduodenectomy; the SMV-PV was resected and reconstructed	Alive without postoperative complications

Charalampoudis et al. [8]	M/74	No	12.7	Body-tail	Microcystic type	No	NA	Attached to the splenic porta and the transverse mesocolon	Distal pancreatectomy with splenectomy	Alive without postoperative complications

Dikmen et al. [9]	F/64	Abdominal pain	15.5	Head	Microcystic type	No	NA	Compression of the right and left PV, inferior vena cava, left PV, and SMA	Whipple procedure	Alive without postoperative complications

Kawaguchi et al. [10]	F/58	Abdominal bloating	20.0	Body	Macrocystic type	No	NA	Compression of the middle part of the gastric body and main pancreatic duct in the tail of the pancreas	Distal pancreatectomy with splenectomy	NA

Dokmak et al. [11]	F/33–66	Pain and fullness in the right subcostal area (*n* = 3), palpable mass (*n* = 3), signs of gastric outlet obstruction (*n* = 1), and cholestasis without jaundice	12.0, 13.0, and 14.0	Head (*n* = 3)	Macrocystic type (*n* = 3)	NA (*n* = 3)	NA (*n* = 3)	NA (*n* = 3)	Laparoscopic fenestration (*n* = 3), and one patient needed pancreatectomy	Bile duct injury in one patient, pancreatic fistula in another patient At the last follow-up (13, 21, and 26 months), all 3 patients were symptom-free

Liu et al.	F/65	Abdominal bloating and vomiting	15.3	Body-tail	Microcystic type	Yes	Lack of abundant feeding arteries (SA and DPA) and draining veins (drainage into the SV)	Encasement or compression of the left RV, the SA and, SV and adherence to the posterior gastric wall	Distal pancreatectomy with splenectomy	No postoperative complications and survived after 14 months of follow-up

Liu et al.	M/67	Acid reflux with abdominal bloating and pain	14.8	Body-tail	Microcystic type	Yes	Abundant feeding arteries (SA) and draining veins (drainage into the SV and the SMV)	Encasement of the SA and SV; gastric vein varices, transverse mesocolon adhesions	Distal pancreatectomy with splenectomy and omentum resection	Postoperative infection and fluid accumulation in the surgical area; survived after 49 months of follow-up

Liu et al.	M/48	Abdominal pain and bloating	10.2	Body-tail	Microcystic type	No	Abundant feeding artery (SA) and draining veins (drainage into the SMV and the SV)	Compression of the left RV and the SV	Distal pancreatectomy with preserving spleen	Mild postoperative pancreatic fistula, survived after 45 months of follow-up

Liu et al.^**∗**^	F/68	Abdominal bloating, palpable mass	16.5	Head	Mix-type	Yes	Lack of abundant feeding artery (GDA) and draining veins (drainage into the SMV)	Encasement and compression of the GDA, the PV, the SMV, and the CBD	Pancreaticoduodenectomy, repair of the injured portal vein	No postoperative complications and survived after 24 months of follow-up

Liu et al.	F/63	Abdominal pain	11.2	Body-tail	Microcystic type	Yes	Abundant feeding artery (SA) and draining veins (drainage into the SMV and the SV)	Encasement and compression of the SA and SV and adherence to the posterior gastric wall and the transverse colon	Distal pancreatectomy withsplenectomy and partial resection of the transverse colon	No postoperative complications and survived after 17 months of follow-up

Liu et al.	M/54	Abdominal bloating	10.5	Body-tail	Microcystic type	Yes	Lack of abundant feeding artery (SA) and draining veins (drainage into the SMV and the SV)	Encasement and compression of the SA and SV and gastric vein varices	Distal pancreatectomy with splenectomy	No postoperative complications and survived after 8 months of follow-up

Note: Y, years; F, female; M, male; PV, portal vein; SMV, superior mesenteric vein; NA, not available; GDA, gastroduodenal artery; RGA, right gastric artery, SA, splenic artery; DPA, dorsal pancreatic artery; IPDA, inferior pancreaticoduodenal arteries; SMA, superior mesenteric artery; IVC, inferior vena cava; SV, splenic vein; RV, renal vein; CBD, common bile duct.

^*∗*^This patient had concurrent gastric stromal tumor and rectal adenocarcinoma.
